# Whole-lesion-aware network based on freehand ultrasound video for breast cancer assessment: a prospective multicenter study

**DOI:** 10.1186/s40644-025-00892-y

**Published:** 2025-06-16

**Authors:** Jie Han, Yuanjing Gao, Ling Huo, Dong Wang, Xiaozheng Xie, Rui Zhang, Mengsu Xiao, Nan Zhang, Meng Lei, Quanlin Wu, Lu Ma, Chao Sun, Xinyi Wang, Lei Liu, Shuzhen Cheng, Binghui Tang, Liwei Wang, Qingli Zhu, Yong Wang

**Affiliations:** 1 Department of Ultrasound, National Cancer Center/National Clinical Research Center for Cancer/Cancer Hospital, Chinese Academy of Medical Sciences and Peking Union Medical College, Chaoyang District Beijing, 100021 China; 2 Department of Ultrasound, Peking Union Medical College Hospital, Chinese Academy of Medical Sciences and Peking Union Medical College, Dongcheng District, Beijing, 100730 China; 3 Key Laboratory of Carcinogenesis and Translational Research (Ministry of Education/Beijing), Department of Breast Center, Peking University Cancer Hospital & Institute, Beijing, China; 4 Yizhun Medical AI Co., Ltd CN, Beijing, China; 5 University of Science and Technology Beijing, Beijing, China; 6 Center for Data Science, Peking University, Beijing, China; 7 Yuanpei College, Peking University, Beijing, China; 8 Department of Ultrasound, Nanchang People’s Hospital, Nanchang, China; 9 State Key Laboratory of General Artificial Intelligence, School of Intelligence Science and Technology, Peking University, Beijing, China

**Keywords:** Breast neoplasms, Ultrasonography, Video, Diagnosis, Deep learning, Artificial intelligence

## Abstract

**Background:**

The clinical application of artificial intelligence (AI) models based on breast ultrasound static images has been hindered in real-world workflows due to operator-dependence of standardized image acquisition and incomplete view of breast lesions on static images. To better exploit the real-time advantages of ultrasound and more conducive to clinical application, we proposed a whole-lesion-aware network based on freehand ultrasound video (WAUVE) scanning in an arbitrary direction for predicting overall breast cancer risk score.

**Methods:**

The WAUVE was developed using 2912 videos (2912 lesions) of 2771 patients retrospectively collected from May 2020 to August 2022 in two hospitals. We compared the diagnostic performance of WAUVE with static 2D-ResNet50 and dynamic TimeSformer models in the internal validation set. Subsequently, a dataset comprising 190 videos (190 lesions) from 175 patients prospectively collected from December 2022 to April 2023 in two other hospitals, was used as an independent external validation set. A reader study was conducted by four experienced radiologists on the external validation set. We compared the diagnostic performance of WAUVE with the four experienced radiologists and evaluated the auxiliary value of model for radiologists.

**Results:**

The WAUVE demonstrated superior performance compared to the 2D-ResNet50 model, while similar to the TimeSformer model. In the external validation set, WAUVE achieved an area under the receiver operating characteristic curve (AUC) of 0.8998 (95% CI = 0.8529–0.9439), and showed a comparable diagnostic performance to that of four experienced radiologists in terms of sensitivity (97.39% vs. 98.48%, *p* = 0.36), specificity (49.33% vs. 50.00%, *p* = 0.92), and accuracy (78.42% vs.79.34%, *p* = 0.60). With the WAUVE model assistance, the average specificity of four experienced radiologists was improved by 6.67%, and higher consistency was achieved (from 0.807 to 0.838).

**Conclusion:**

The WAUVE based on non-standardized ultrasound scanning demonstrated excellent performance in breast cancer assessment which yielded outcomes similar to those of experienced radiologists, indicating the clinical application of the WAUVE model promising.

**Supplementary Information:**

The online version contains supplementary material available at 10.1186/s40644-025-00892-y.

## Background

Female breast cancer stands as a prominent contributor to cancer-related mortality among women globally [1]. In China, the landscape of cancer is in transition, breast cancer has shown a rapid increase in incidence rate among younger generation and an accelerated mortality rate in older populations [2]. Breast ultrasound (US) has gained widespread use owing to its inherent conveniences, radiation-free nature, and efficacy in detecting breast cancer, especially in dense breasts. However, the operator-dependent nature of US often results in a high recall rate and low positive predictive value for biopsies. Particularly in China, where radiologists conduct bilateral whole breast freehand scans and provide real-time on-site assessments, the process proves to be time and energy-consuming. Consequently, there is significant value in developing assisted diagnostic tools to alleviate the workload of radiologists and ensure consistent diagnosis across different operators.

In recent years, the rapid growth of artificial intelligence (AI), particularly deep learning (DL), in the realm of ultrasonic imaging has been notable [3–5]. This progress offers a promising avenue to mitigate the inherent operator-dependence associated with US. Numerous studies have explored the application of AI in diagnosing both benign and malignant breast tumors [6–15]. Some AI-based computer-aided diagnosis (CAD) systems have successfully obtained approval of the Food and Drug Administration (FDA) [16, 17]. Despite satisfactory diagnostic performance [14, 18–20], the utilization of these AI products and models remains constrained. Both research and commercial software predominantly rely on static ultrasonic images from various sections or multimodal images (B-mode US, color Doppler US, elastography, contrast-enhanced US, automated breast US) [13, 21–23], notably, the static images used to train AI models are typically meticulously curated by experienced radiologists. S-Detect™, a commercially available CAD system, has emerged as one of the most increasingly used tools for the diagnosis of breast cancer. A multicenter prospective study, conducted across nine medical centers spanning diverse economic statuses and healthcare resources in China, revealed significant differences in S-Detect’s diagnostic performance across study sites, and further indicated that discrepancies in US imaging acquisition proficiency likely contributed to the observed differences in diagnostic outcomes [24]. Studies have also showed that 11.6% −18.1% breast lesions exhibited inconsistent S-Detect outcomes in radial and anti-radial planes [25, 26], and the AUC improved significantly from 0.76 with the cross-planes to 0.84 with the quadri-planes methods [27]. Furthermore, static images may fail to capture the overall morphological characteristics of tumors and their relationship with surrounding structures, potentially leading AI models to misinterpret hyperplastic glandular tissue or ribs as lesions [28]. Additionally, the availability of imaging modes such as elastography or contrast-enhanced US is not consistent in primary medical institutions.

To address these challenges and fully harness information from the whole lesion, we devised a whole-lesion-aware network based on freehand ultrasound video (WAUVE). This innovative approach bypassed the traditional step of manually selecting a key frame image by radiologists, empowering the model to holistically analyze comprehensive information from the entire lesion for breast cancer assessment. We substantiated the superior performance achieved by constructing the model with dynamic videos as opposed to static images. Our study validated diagnostic efficacy and generalizability of the model, and we delved into its potential to assist experienced radiologists.

## Methods

### Development and external validation datasets

We retrospectively collected breast US videos and their corresponding static images from Peking University Cancer Hospital & Institute and Nanchang People's Hospital between May 2020 and August 2022. These cases were primarily from patients who were either recalled after routine screening due to suspicious findings or presented with symptoms requiring further diagnostic evaluation. Not all lesions were initially classified as suspicious or recommended for biopsy at the participating institutions. Some patients were referred after breast lesions were first identified at other facilities, and a proportion of BI-RADS 2 and 3 lesions were included to ensure the representation of benign cases and better reflect real-world distributions. Breast US was performed by qualified radiologists using high-frequency linear transducers under the machine’s preset condition. In these tertiary hospitals, it is standard clinical practice to save both static images and full-length US video clips during each examination. Videos were saved in AVI format and static images in JPEG. No mandatory requirements for experience or skill level of radiologists who performed US examinations were applied in this study. We only emphasized the integrity of breast lesion US videos, which were required to contain the entire lesion area in an arbitrary scanning direction and speed based on individual habits to recapitulate real-world routine clinical workflows. Meanwhile, for each lesion, two key frames were selected as the static images: (1) in cases where no suspicious malignant features were identified, the key frames were chosen as the largest cross-section of the lesion and a second orthogonal view; (2) for lesions with suspicious malignant characteristics, radiologists were allowed to select two views showing such features, regardless of orientation. Suspicious malignant features referred to irregular shape, non-parallel orientation, non-circumscribed margin, posterior acoustic shadowing, and echogenic halo, as defined by the BI-RADS lexicon.

**Fig. 1:**
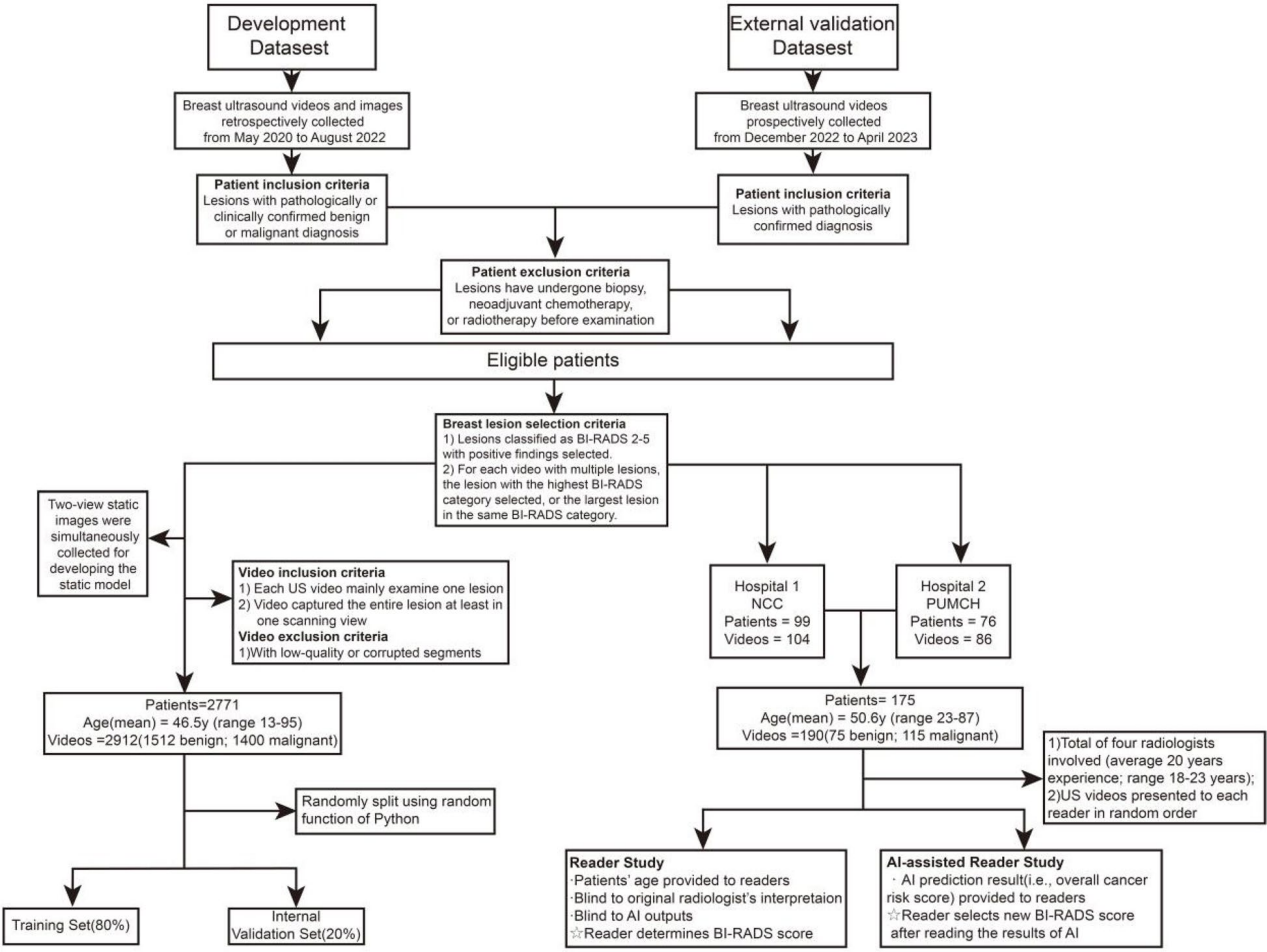
Overview of the retrospective development and prospective external validation dataset workflows. The development dataset was collected in two hospitals before August 2022, while the external validation dataset was collected in two other hospitals after development of the deep learning model. Abbreviations: NCC, National Cancer Center; PUMCH, Peking Union Medical College Hospital

To further evaluate model performance and generalizability, we prospectively collected 190 breast US videos from National Cancer Center (NCC) and Peking Union Medical College Hospital (PUMCH) between December 2022 and April 2023. These data were collected after the development of the model. Details of the inclusion and exclusion criteria of the development and external validation datasets are presented in Fig. 1. For benign lesions without histopathologic results, a clinically confirmed diagnosis was established based on at least two years of imaging follow-up with no change in size or imaging characteristics, as per standard clinical practice. In the prospective dataset, all lesions had histopathologic confirmation. A small number of these were classified as BI-RADS 3 on imaging. In these cases, biopsy was performed due to strong patient preference, driven by high risk factors, anxiety or cosmetic concerns—a practice that reflects common clinical scenarios in China.

All videos and images processed for this study were de-identified before transferring to study investigators.

### Dynamic WAUVE model development

**Fig. 2:**
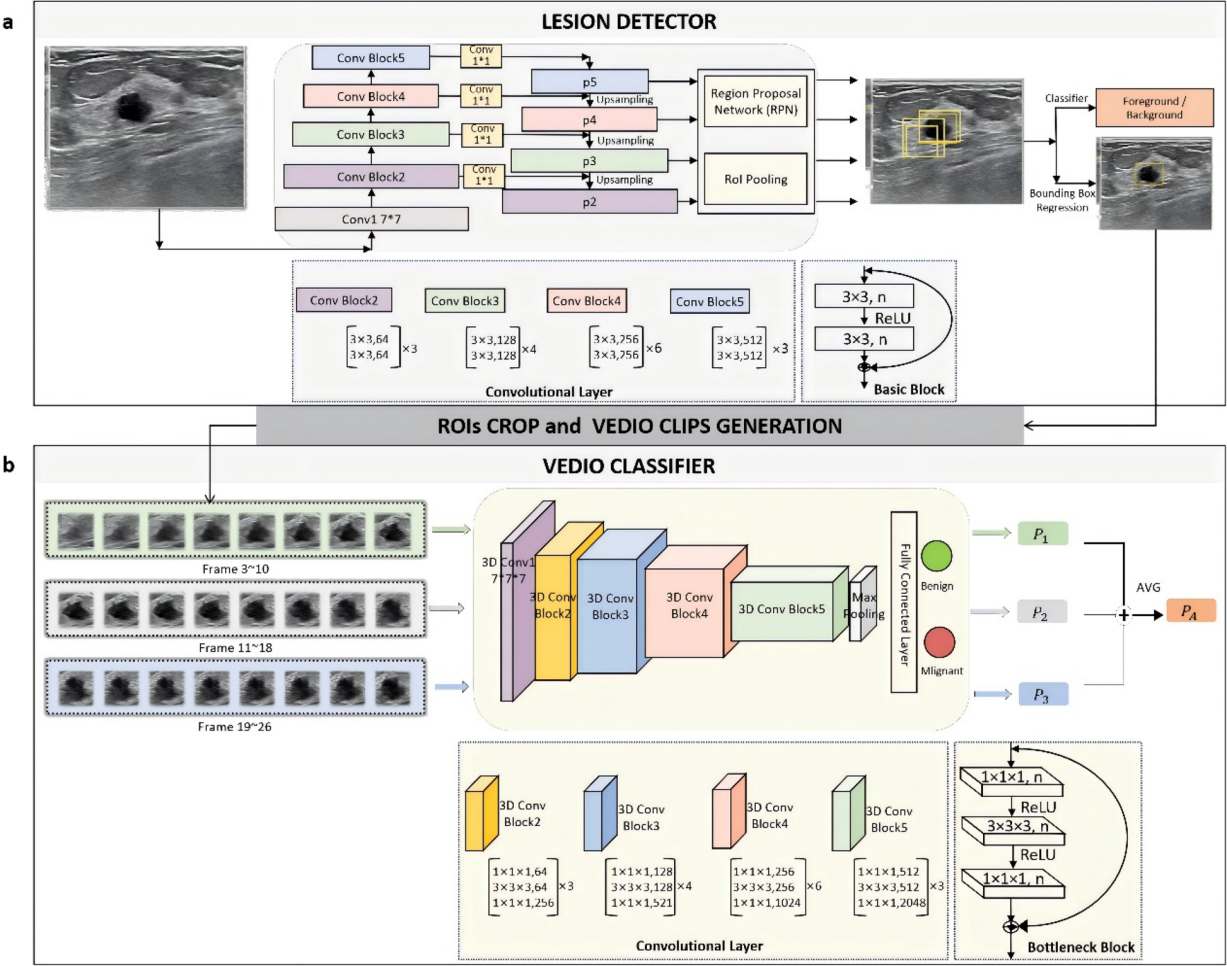
The schematic architecture of the whole-lesion-aware network for freehand ultrasound video(WAUVE). For a given ultrasound video, (**a**) the Faster R-CNN with ResNet-34 backbone firstly extracts regions-of-interest (ROIs) from each frame. Then ROIs are cropped to generate video clips for each video, where each adjacent eight frames are grouped as a video clip. Subsequently, (**b**) Inflated 3D ConvNet (I3D) with ResNet-50 backbone performs malignancy probability prediction for each video clip. Finally, the overall cancer risk score is outputted in the form of the average of the results of all video clips

Our WAUVE model for breast cancer risk prediction used US videos as input and generated a cancer risk score between 0 and 1 to predict the risk of cancer existence for each video. The entire pipeline of the WAUVE model is presented in Fig. 2. The model comprised two components:

1. Lesion detector performed lesion detection frame-by-frame to select region-of-interest (ROI) for further assessment. We adopted the Faster R-CNN [29] with ResNet-34 backbone [30] as the lesion detection network, and the feature pyramid network (FPN) [31] was used to enhance the lesion detection capabilities on different scales.
2. Video classifier predicted the cancer risk score by aggregating the predictions of a series of video clips with eight frames. To create the video clips, images within the ROI regions were firstly cropped. Then, we grouped each adjacent eight frames sequentially in chronological order in a track and predicted the cancer risk score for each clip. Tracks less than eight frames and extra ROIs at the end of the track that were insufficient to form the eight-frame clip were eliminated from calculations of cancer risk score for simplicity. The video classifier was instantiated using the Inflated 3D ConvNet (I3D) [32] with ResNet-50 backbone [30] in WAUVE.

More details of the model development are described in Additional file 1. All models were run using the GPU server with Intel Xeon CPU E5-2680 v4 @ 2.40 GHz and 8 NVIDIA TITAN RTX GPUs. PyTorch 1.10.1 was employed in our experiments. Detectron2 and SlowFast frameworks were adopted to implement the lesion detector and video classifier, respectively.

To illustrate the informative regions in each video and improve the interpretability of our AI model, a global average pooling (GAP) [33] layer was added after the last convolutional layer in the video classifier to generate heatmaps.

### Comparison with other DL models

We developed two other DL models based on different classifier backbones for comparison: a static model using 2D-ResNet50, and a dynamic model using TimeSformer. The detailed descriptions of the two models’ construction are shown in Additional file 1. We tested the static model on three input patterns: test on the single key frame, test on the two key frames (transverse and longitudinal views), and test on all ROI images of video. In contrast, the entire dynamic video was directly fed into our WAUVE model and the TimeSformer model to generate the final malignancy score.

### Reader and AI-assisted reader studies

To compare the performance between WAUVE and experienced radiologists, and to analyze the auxiliary value of the WAUVE for experienced radiologists (18 to 23 years of clinical experience in breast ultrasound), we conducted a two-part reader-study on the external validation dataset. In the first part, the four radiologists (YW, QZ, RZ, MX) who were blinded to the pathological diagnosis independently evaluated and provided a Breast Imaging Reporting and Data System (BI-RADS) score for each breast US video in isolation. In the second part, the radiologists reconsidered the BI-RADS score after referring to the malignancy score of the AI model.

### Statistical analysis

Continuous variables with a normal distribution are expressed as the mean ± standard deviation and were analyzed using an unpaired t-test. Non-normally distributed continuous variables are presented as medians and ranges which were analyzed using the Wilcoxon rank sum test. Categorial variables are expressed as frequencies and percentages which were analyzed using χ^2^ test. The area under the receiver operating characteristic curve (AUC), sensitivity, specificity, positive predictive value (PPV), and negative predictive value (NPV), accuracy, and F1 score of WAUVE, 2D-ResNet50, and TimeSformer models were calculated in the internal validation set. McNemar’s test was used to compare the sensitivity, specificity, and accuracy. The χ^2^ test was used to compare the PPV and NPV. In the reader study, the diagnostic performance of WAUVE was expressed as the area under the ROC curve (AUC), while radiologists’ performance was represented as individual operating points using BI-RADS 3 versus 4a and above as the binary threshold (i.e., BI-RADS ≥ 4a considered malignant). The Delong’s test was used to compare AUCs. The agreement between two radiologists was qualified using Kappa values, while interclass correlation coefficients (ICCs) were used to assess the agreement between four radiologists. *p* < 0.05 was considered a statistically significant difference. All statistical analyses were performed using SPSS (version 26; IBM), MedCalc (version 20.1.0; MedCalc software), and Python 3.6.5.

## Results

### Dataset characteristics

Table 1 lists the detailed patient demographics, breast lesion characteristics, and US machine vendors. Each video and lesion had a one-to-one correspondence. Among the 2912 videos in the development dataset, 2273 videos from 2203 patients were collected from Nanchang People's Hospital, while 639 videos from 568 patients were collected from Peking University Cancer Hospital & Institute. The frame rate of videos was predominantly 30 or 50, and frame numbers in all videos ranged from 12 to 4297. Additionally, the frame size of each video is 1920 × 1080.

**Table 1:** Patient demographics and breast lesion characteristics of the retrospective training, internal validation dataset and prospective external validation dataset

Characteristics, unit	Retrospective dataset			Prospective dataset		
	Training set	Internal Validation set	*p* value	NCC	PUMCH	*p* value
Patients, N	2204	567		99	76	
Age,years, mean ± SD	46.5 ± 12.3	46.4 ± 13.4	0.99	52.2 ± 13.6	48.4 ± 14.5	0.08
< 50 years old, N (%)	1353 (61.4%)	350 (61.7%)		42 (42.4%)	46 (60.5%)	
≥ 50 years old, N (%)	851 (38.6%)	217 (38.3%)		57 (57.6%)	30 (39.5%)	
Videos/lesions, N	2336	576		104	86	
Lesion size, mm, median (range)	18.4 (2.9–93.0)	19.3 (3.9–87.0)	0.009	16.0 (5.0–48.0)	16.0 (5.0–48.0)	0.45
≤ 10 mm, N (%)	377 (16.1%)	87 (15.1%)		16 (15.4%)	24 (27.9%)	
> 10 mm, N (%)	1959 (83.9%)	489 (84.9%)		88 (84.6%)	62 (72.1%)	
BI-RADS category^a^			0.41			0.000
2, N (%)	185 (7.9%)	52 (9.0%)		0	1 (1.2%)	
3, N (%)	399 (17.1%)	83 (14.4%)		2 (2.0%)	24 (27.9%)	
4A, N (%)	598 (25.6%)	142(24.7%)		25 (24.0%)	18 (20.9%)	
4B,N(%)	493 (21.1%)	117 (20.3%)		25 (24.0%)	6 (7.0%)	
4C, N (%)	510 (21.8%)	137 (23.8%)		26 (25.0%)	26 (30.2%)	
5, N (%)	151 (6.5%)	45 (7.8%)		26 (25.0%)	11 (12.8%)	
Machine			0.70			
GE, N (%)	1603 (68.6%)	379 (65.8%)		0	0	
Siemens, N (%)	273 (11.7%)	71 (12.3%)		0	0	
Philips, N (%)	107 (4.6%)	27 (4.7%)		104 (100.0%)	86 (100.0%)	
Esaote, N (%)	261 (11.2%)	76 (13.2%)		0	0	
Others^b^, N (%)	92 (3.9%)	23 (4.0%)		0	0	
Lesion type			0.45			0.08
Invasive ductal carcinoma, N (%)	914 (39.1%)	236 (41.0%)		54 (51.9%)	34 (39.5%)	
Ductal carcinoma in situ, N (%)	93 (4.0%)	30 (5.2%)		8 (7.7%)	6 (7.0%)	
Other malignant tumors^c^, N (%)	104 (4.5%)	22 (3.8%)		9 (8.7%)	4 (4.7%)	
Fibroadenoma, N (%)	644 (27.6%)	139 (24.1%)		18 (17.3%)	19 (22.1%)	
Intraductal papilloma, N (%)	71 (3.0%)	17 (3.0%)		3 (2.9%)	11 (12.7%)	
Other benign tumors^d^, N (%)	510 (21.8%)	132 (22.9%)		12 (11.5%)	12 (14.0%)	

*Abbreviations:* *N* number, *SD* standard deviation, *NCC* National Cancer Center, *PUMCH* Peking Union Medical College Hospital. ^a^BI-RADS category was extracted from sonographic reports before biopsy or surgery but not the reader study results. ^b^Includes Samsung, Canon, Sonoscape, and Mindray. ^c^Includes invasive lobular carcinoma, mucinous carcinoma, carcinoma with medullary features, invasive micropapillary carcinoma, solid papillary carcinoma, apocrine carcinoma, and malignant without specific type. ^d^Includes adenosis, hyperplasia, tubular adenoma, benign phyllodes tumor, cyst, inflammation, and clinically diagnostic benign (in retrospective dataset) with at least 2-year follow-up.

*Abbreviations*: *N* number, *SD* standard deviation, *NCC* National Cancer Center, *PUMCH* Peking Union Medical College Hospital          ^a^ BI-RADS category was extracted from sonographic reports before biopsy or surgery but not the reader study results. ^b^ Includes Samsung, Canon, Sonoscape, and Mindray. ^c^ Includes invasive lobular carcinoma, mucinous carcinoma, carcinoma with medullary features, invasive micropapillary carcinoma, solid papillary carcinoma, apocrine carcinoma, and malignant without specific type. ^d^ Includes adenosis, hyperplasia, tubular adenoma, benign phyllodes tumor, cyst, inflammation, and clinically diagnostic benign (in retrospective dataset) with at least 2-year follow-up.

### Comparison of the diagnostic performance of WAUVE model with other DL models

**Fig. 3:**
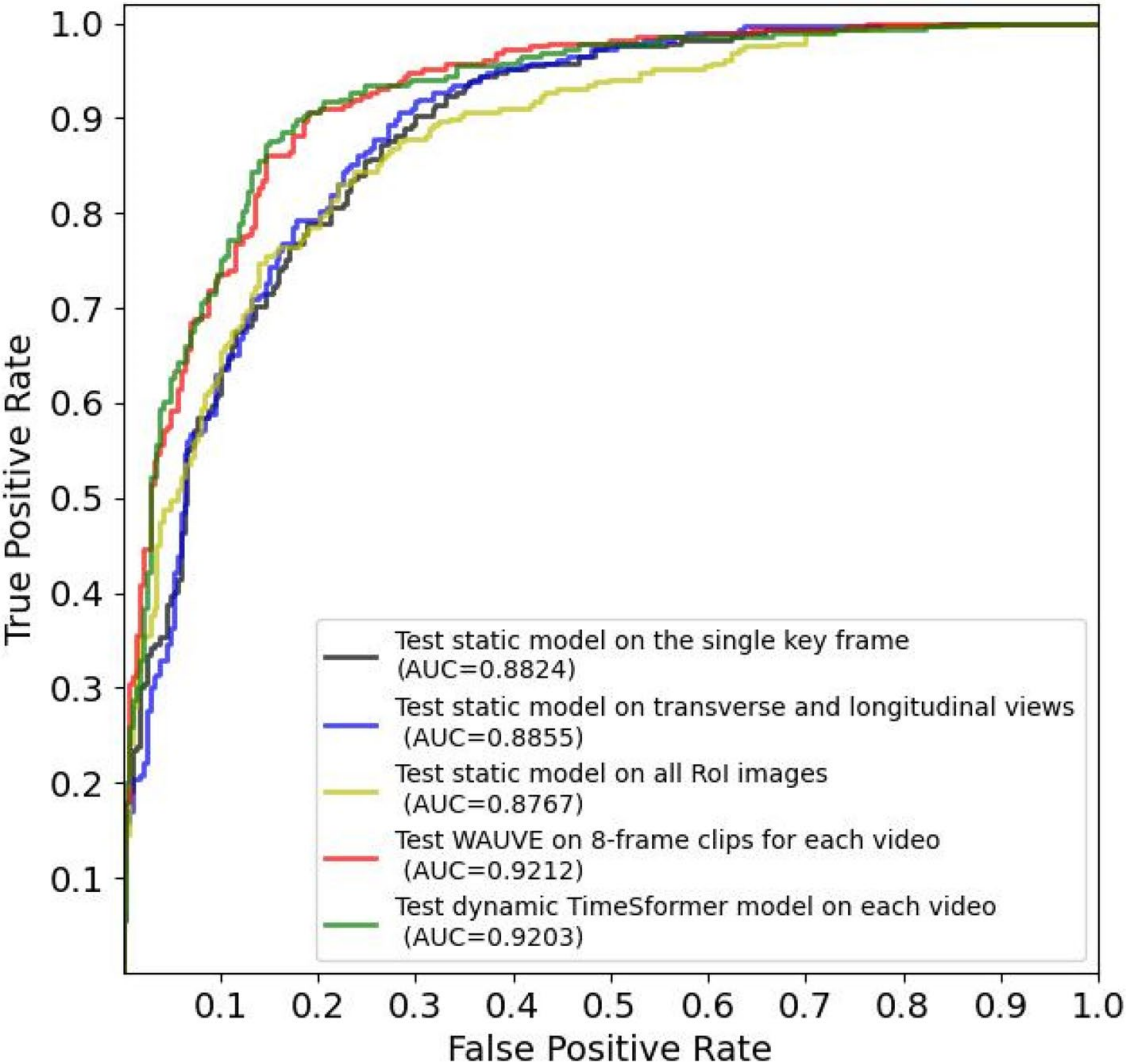
Receiver operating characteristic curves for different DL models in the internal validation dataset. Abbreviations: AUC, area under the receiver operating characteristic curve; DL, deep learning

The WAUVE model achieved an excellent diagnostic performance in the training set with an AUC of 0.9745 (95%CI = 0.9692–0.9794). In the internal validation set, the WAUVE achieved an AUC of 0.9212, which was superior to that obtained by the static 2D-ResNet50 model tested on the single key frame (AUC: 0.8824,* p* = 0.002), two key frames (AUC: 0.8855, *p* = 0.003), and all ROI images of the video (AUC: 0.8767, *p* = 0.000), and was similar to that of the dynamic TimeSformer model (AUC: 0.9203, *p* = 0.92). More detailed metrics are shown in Fig. 3 and Table 2.

**Table 2:** Evaluation and comparison of the diagnostic performance of the different DL model

	AUC (95%CI)	*p* value	SENS (%,95%CI)	*p* value	SPEC (%,95%CI)	*p* value	PPV (%,95%CI)	*p* value	NPV (%,95%CI)	*p* value	ACC (%,95%CI)	*p* value	F1
WAUVE	0.9212 (0.8992–0.9419)	–-	86.16 (81.63–89.92)	–-	85.37 (80.74–89.25)	–-	85.57 (81.71–88.73)	–-	85.97 (82.07–89.13)	–-	85.76 (82.64–88.52)	–-	0.865
2D-ResNet50													
Input single key frame	0.8824 (0.8553–0.9092)	0.002^*^	85.47 (80.87–89.32)	0.89	75.26 (69.85–80.14)	0.000^*^	77.67 (73.87–81.06)	0.01^*^	83.72 (79.42–87.27)	0.47	80.38 (76.90–83.55)	0.02^*^	0.821
Input two key views	0.8855 (0.8580–0.9119)	0.003^*^	90.66 (86.70–93.75)	0.08	71.78 (66.19–76.91)	0.000^*^	76.39 (72.82–79.61)	0.004^*^	88.41 (84.10–91.67)	0.41	81.25 (77.82–84.36)	0.01^*^	0.829
Input all ROI frames	0.8767 (0.8509–0.9056)	0.000^*^	83.05 (78.21–87.19)	0.27	78.05 (72.81–82.70)	0.004^*^	79.21 (75.27–82.66)	0.004^*^	82.05 (77.86–85.60)	0.21	80.56 (77.08–83.71)	0.004^*^	0.812
TimeSformer	0.9203 (0.8969–0.9415)	0.92	87.20 (82.79–90.82)	0.76	85.37 (80.74–89.25)	1.00	85.71 (81.89–88.84)	0.96	86.88 (83.00–89.98)	0.75	86.29 (83.20–88.99)	0.81	0.867

*Abbreviations:* *DL* Deep learning, *WAUVE *Whole-lesion-aware network based on freehand ultrasound video, *AUC *Area under the receiver operating characteristic curve, *SENS *Sensitivity, *SPEC* Specificity, *PPV* Positive predictive value, *NPV* Negative predictive value, *ACC* Accuracy, *ROI*  Region-of-interest

*p value shows statistical difference

### Performance of the WAUVE in the external validation set

The WAUVE yielded a satisfactory performance in the external validation set, with an AUC of 0.8998 (95%CI = 0.8529–0.9439), a sensitivity of 97.39% (95%CI = 92.57–99.46%), and a specificity of 49.33% (95%CI = 37.59–61.14%). We further tested the WAUVE model in different hospital, patient age, lesion size, and BI-RADS category subgroups (Table 3).

**Table 3:** Performance of the WAUVE model on different subgroups of the external validation dataset

	AUC (95%CI)	*p* value	SENS (%,95%CI)	*p* value	SPEC (%,95%CI)	*p* value	PPV (%,95%CI)	*p* value	NPV (%,95%CI)	*p* value	ACC (%,95%CI)	*p* value
Different hospital												
PUMCH	0.8815 (0.8179,0.9594)	0.48	97.73 (87.98,99.94)	0.84	42.86 (27.72,59.04)	0.21	64.18 (57.87,70.03)	0.008^*^	94.74 (71.54,99.23)	0.62	70.93 (60.14,80.23)	0.023^*^
NCC	0.9168 (0.8615,0.9921)		97.18 (90.19,99.66)		57.58 (39.22,74.52)		83.13 (76.77,88.02)		90.48 (70.14,97.46)		84.62 (76.22,90.94)	
Patient age(y)												
< 50	0.9040 (0.8350,0.9571)	0.72	95.35 (84.19,99.43)	0.29	53.70 (39.61,67.38)	0.23	62.12 (54.98,6.77)	0.002^*^	93.55 (78.56,98.29)	0.64	72.17 (62.14,80.79)	0.033^*^
≥ 50	0.8876 (0.8211,0.9538)		98.61 (92.50,99.97)		38.10 (18.11,61.57)		84.52 (79.59,88.44)		88.89 (51.45,98.37)		84.95 (76.03,91.52)	
Lesion size(mm)												
≤ 10	0.9423 (0.8199,0.9913)	0.17	100.00 (76.84,100.00)	1.00	53.85 (33.38,73.41)	0.63	53.85 (43.51,63.86)	0.012^*^	100.00	0.54	70.00 (53.47,83.44)	0.19
> 10	0.8804 (0.8174,0.9276)		97.03 (91.56,99.38)		46.94 (32.53,61.73)		79.03 (74.29,83.10)		88.46 (70.75,96.05)		80.67 (73.43,86.65)	
BI-RADS category												
BI-RADS 2–3	0.8889 (0.7084,0.9765)	1.00	100.00 (29.24,100)	1.00	62.50 (40.59,81.20)	0.14	25.00 (16.59,35.84)	0.000^*^	100.00	0.28	66.67 (46.04,83.48)	0.12
BI-RADS 4–5	0.8894 (0.8308,0.9331)		97.32 (92.37,99.44)		43.14 (29.35,57.75)		78.99 (74.71,82.71)		88.00 (69.69,95.90)		80.37 (73.43,86.17)	

*Abbreviations*: *WAUVE* Whole-lesion-aware network based on freehand ultrasound video, *PUMCH* Peking Union Medical College Hospital, *NCC* National Cancer Center, *BI-RADS* Breast imaging reporting and data system, *AUC* Area under the receiver operating characteristic curve, *SENS* Sensitivity, *SPEC* specificity, *PPV* Positive predictive value, *NPV* Negative predictive value, *ACC* Accuracy, *CI* Confidence interval

^*^ *p* value shows statistical difference

### AI model performance comparable to experienced radiologists

**Fig. 4:**
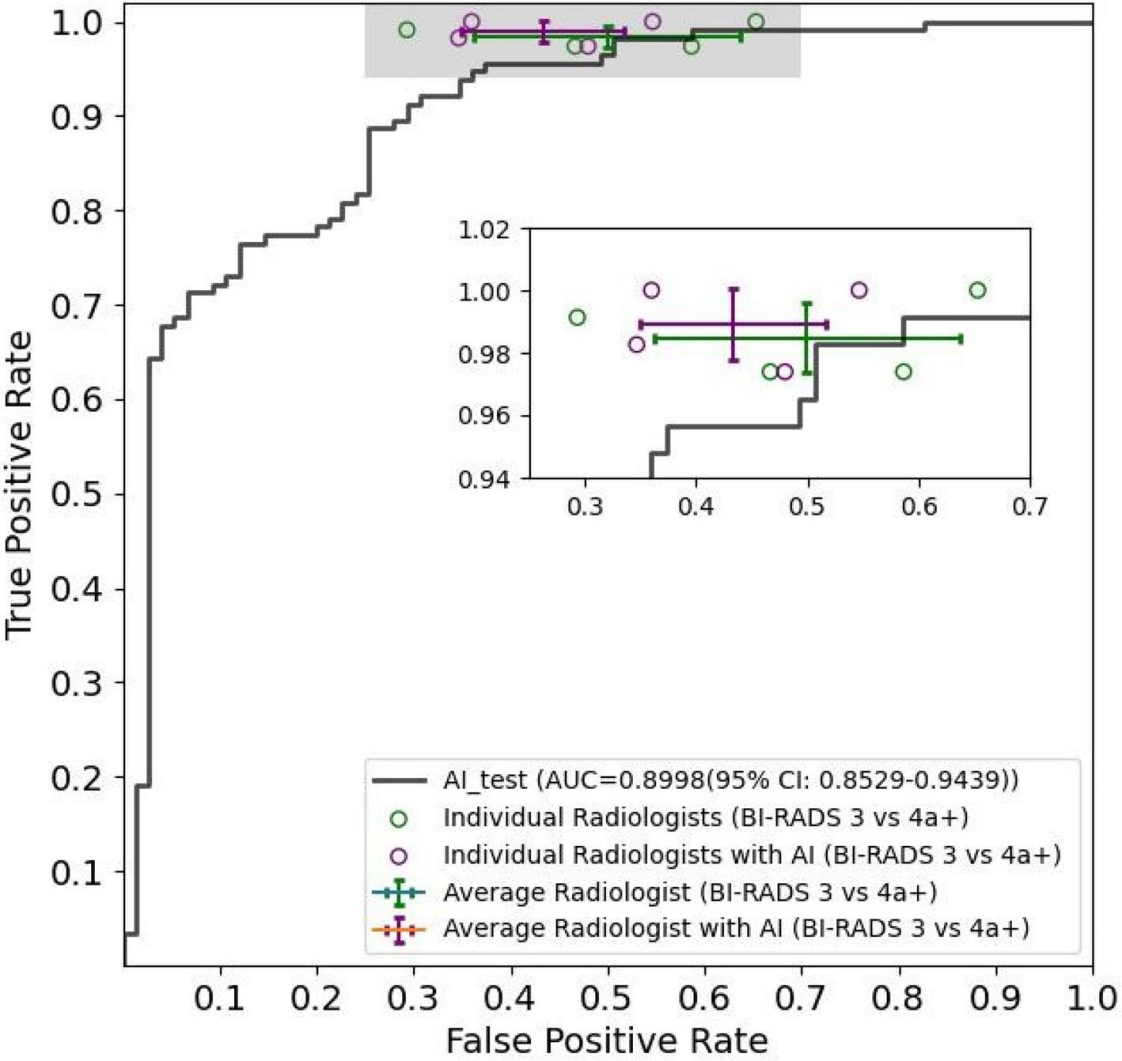
Diagnostic performance comparison between WAUVE model and four radiologists. Receiver operating characteristic (ROC) curves and points are used to depict the performance of the model and four radiologists, respectively. Additionally, the mean performance of four radiologists is presented by error bars with 95% confidence intervals (CIs), which were calculated based on 9999 bootstraps of the data. Green and purple points represent the performance of radiologists without and with AI assistance, respectively

For the WAUVE model, a cancer risk score threshold of 0.2102 was used to dichotomize predictions. This threshold was selected to match the sensitivity level of the average performance of the four participating radiologists, enabling a fair and clinically meaningful comparison. WAUVE achieved comparable performance to the average performance of four radiologists, whereby the mean value in the error bars (green) approximates the point on the ROC curve (Fig. 4). A comparison of the diagnostic performance of WAUVE and the four radiologists is displayed in Table 4.

**Table 4:** Diagnostic performance of the AI system, radiologists without assistance and AI-assisted radiologists

	SENS (%,95%CI)	*p* value	SPEC (%,95%CI)	*p* value	PPV (%,95%CI)	*p* value	NPV (%,95%CI)	*p* value	ACC (%,95%CI)	*p* value
AI	97.39 (92.57,99.46)	–-	49.33 (37.59,61.14)	–-	74.67 (70.17,78.69)	–-	92.50 (79.77,97.47)	–-	78.42 (71.89,84.05)	–-
Radiologists without assistance										
R1	97.39 (92.57,99.46)	1.00^*^	53.33 (41.45,64.95)	0.70^*^	76.19 (71.49,80.33)	0.76^*^	93.02 (81.06,97.65)	0.93^*^	80.00 (73.60,85.44)	0.73^*^
R2	99.13 (95.25,99.99)	0.63^*^	70.67 (59.02,80.62)	0.005^*^	83.82 (78.47,88.05)	0.058^*^	98.15 (88.22,99.73)	0.18^*^	87.90 (82.39,92.17)	0.003^*^
R3	97.39 (92.57,99.46)	1.00^*^	41.33 (30.08,53.30)	0.35^*^	71.80 (67.74,75.527)	0.57^*^	91.18 (76.61,97.02)	0.84^*^	75.26 (68.50,81.22)	0.39^*^
R4	100.00 (96.84,100.00)	0.25^*^	34.67 (24.04,46.54)	0.035^*^	70.12 (66.56,73.46)	0.37^*^	100.00 (100.00,100.00)	0.16^*^	74.21 (67.38,80.27)	0.17^*^
AVG	98.48 (96.89,99.39)	0.36^*^	50.00 (44.20,55.80)	0.92^*^	75.12 (72.94,77.19)	0.86^*^	95.54 (91.06,97.83)	0.26^*^	79.34 (76.29,82.17)	0.60^*^
Radiologists + AI										
R1	100.00 (96.84,100.00)	0.25^#^	64.00 (52.09,74.77)	0.022^#^	80.99 (75.90,85.21)	0.32^#^	100.00 (100.00,100.00)	0.06^#^	85.79 (80.00,90.42)	0.003^#^
R2	98.26 (93.86,99.79)	1.00^#^	65.33 (53.46,75.96)	0.39^#^	81.30 (76.09,85.58)	0.58^#^	96.08 (86.00,98.99)	0.53^#^	85.26 (79.41,89.98)	0.27^#^
R3	97.39 (92.57,99.46)	1.00^#^	52.00 (40.15,63.69)	0.008^#^	75.68 (71.04,79.78)	0.44^#^	92.86 (80.65,97.59)	0.79^#^	79.47 (73.03,84.98)	0.008^#^
R4	100.00 (96.84,100.00)	1.00^#^	45.33 (33.79,57.25)	0.039^#^	73.72 (69.54,77.51)	0.48^#^	100.00 (100.00,100.00)	0.30^#^	78.42 (71.89,84.05)	0.039^#^
AVG	98.91 (97.48,99.65)	0.63^#^	56.67 (50.85,62.35)	0.003^#^	77.78 (75.46,79.94)	0.28^#^	97.14 (93.40,98.79)	0.45^#^	82.24 (79.33,84.89)	0.002^#^

*Abbreviations*: *AVG* Average, *SENS* Sensitivity, *SPEC* Specificity, *PPV* Positive predictive value, *NPV* Negative predictive value, *ACC* Accuracy, *CI* Confidence interval

^*^p values refer to the AI system; ^#^*p* values refer to radiologists without assistance

### AI-assisted radiologists achieve better performance

With the assistance of the WAUVE, the average diagnostic performance of the four radiologists achieved better specificity (56.67% vs. 50.00%, *p* = 0.003) and accuracy (82.24% vs. 79.34%, *p* = 0.002). More detailed metrics are listed in Table 4.

Table 5 shows each radiologist’s BI-RADS adjustments after considering the prediction result of WAUVE. In general, the radiologists preferred to upgraded more BI-RADS categories in malignant lesions, and downgrade more BI-RADS categories in benign lesions. In reference to the binary clinical strategy of follow-up or biopsy (BI-RADS 3 versus 4a +), we observed that four radiologists accurately downgraded cases 31 times, that is, unnecessary biopsies were avoided from benign lesions.

**Table 5:** BI-RADS category adjustments of four radiologists after the AI-assisted reader study

Adjustment	No change(n)	Upgrade(n)						Downgrade(n)							
		Total	3 → 4A	4A → 4B	4B → 4C	3 → 4B	4C → 5	Total	4A → 3	4B → 4A	4C → 4B	4C → 4A	3 → 2	5 → 4C	4B → 3
R1-Benign (75)	59	3	1	1	1	0	0	13	9	2	0	1	1	0	0
R1-Malignant (115)	106	7	2	0	4	1	0	2	0	0	2	0	0	0	0
R2-Benign (75)	60	9	7	0	1	1	0	6	4	1	0	1	0	0	0
R2-Malignant (115)	73	25	0	2	9	0	14	17	1	6	7	0	0	3	0
R3-Benign (75)	67	0	0	0	0	0	0	8	7	0	0	0	0	0	1
R3-Malignant (115)	106	5	0	1	4	0	0	4	0	0	3	0	0	1	0
R4-Benign (75)	54	5	2	3	0	0	0	16	10	5	0	0	0	1	0
R4-Malignant (115)	84	17	0	5	10	0	2	14	0	4	7	0	0	3	0

**Fig. 5:**
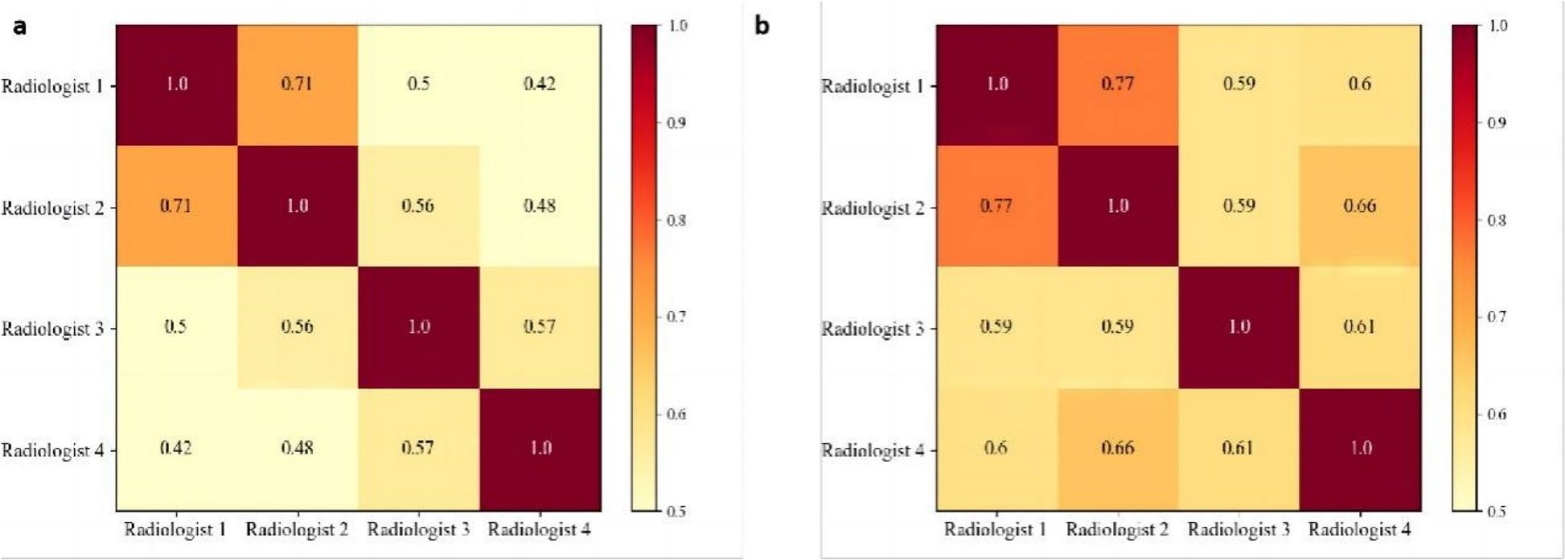
Consistency between different radiologists before (**a**) and after (**b**) AI assistance. Based on the color bar on the right, the color block darkens with increasing consistency, and the number in the color block area represents the Kappa value between two corresponding radiologists. With AI assistance, the consistency between radiologists was higher than that before AI assistance, and the Kappa values between different radiologists were all improved to different degrees

Changes in diagnostic consistency between radiologists before and after using the WAUVE are presented in Fig. 5. With the WAUVE assistance, the inter-class variation between two radiologists was reduced, leading to more consistent diagnostic results (with ICC from 0.807 to 0.838).

Based on the independent diagnosis time of radiologists, we defined evaluation time as the overall duration of diagnosis minus the duration of primary videos. Subsequently, we divided all videos into three groups: −10.45–12.67 s, 13.03–26.03 s, and 26.08–95.49 s. In all three groups, compared with the results of radiologists independently, the specificity of radiologists with the WAUVE assistance increased by 5% to 8%, especially in the most time-consuming group (*p* = 0.027), while the sensitivity remained stable (Additional file 1).

### Qualitative illustration and heatmap interpretation of the WAUVE model

**Fig. 6:**
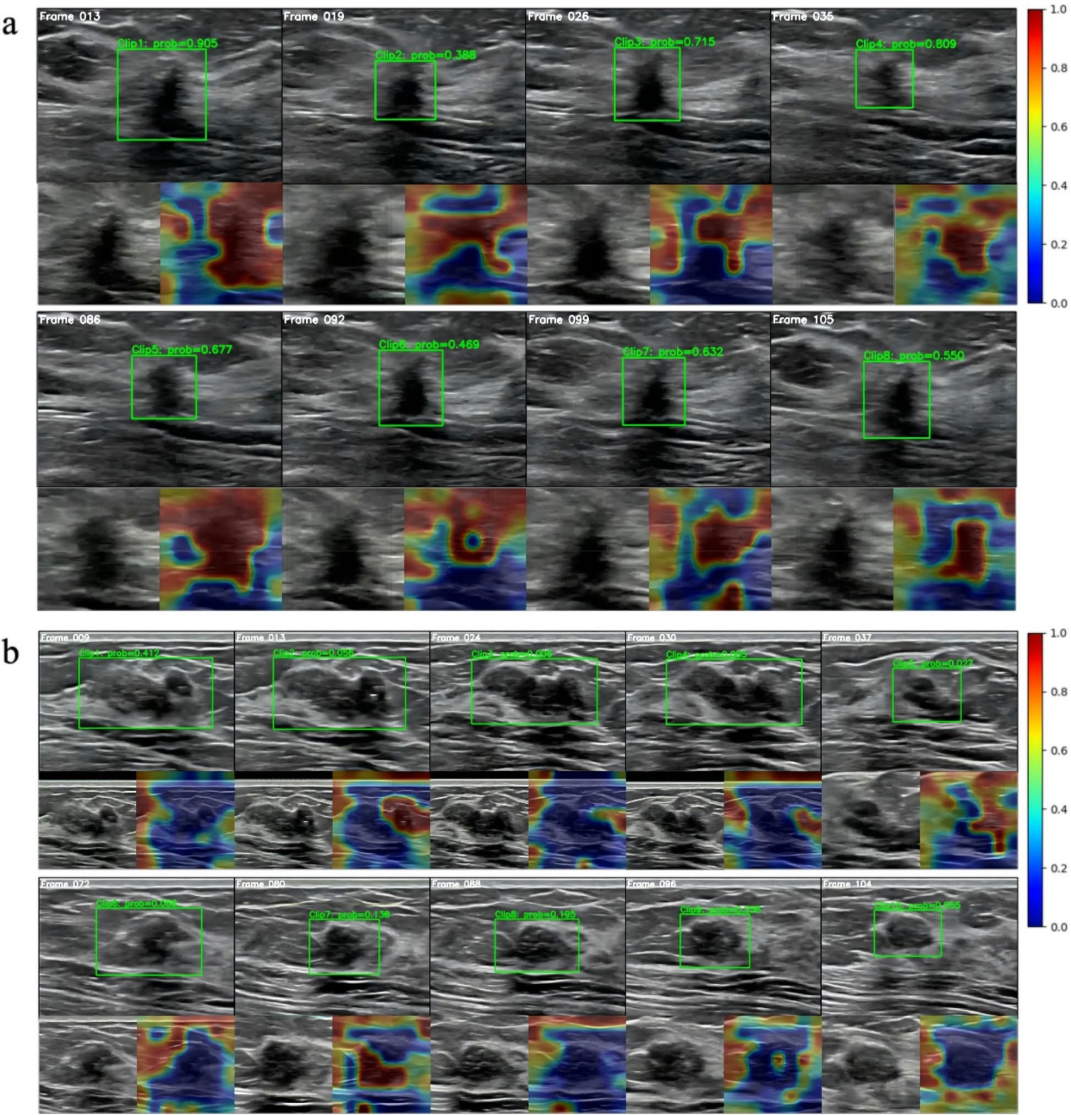
Examples of Qualitative illustration and heatmap illustration of our WAUVE model. We present representative frames from the input video and the corresponding heatmaps. The green bounding boxes represent the detected ROI in each frame. For each ROI, the cancer risk score of the video clip (Clip N: prob = X) is presented above the bounding box. In the heatmap, the red regions contribute to high malignancy probability and the blue regions to benign. **a** The WAUVE model obtained an overall cancer risk score of 0.643, with mostly red-colored regions suggesting a malignant lesion. All four radiologists in the reader study classified this lesion as BI-RADS 4 C, and the final histopathological diagnosis was a malignancy. **b** The WAUVE model obtained an overall cancer risk score of 0.111, with mostly blue regions suggesting a benign lesion. One radiologist assigned this lesion as BI-RADS 4a and another as 4b, indicating mild to moderate suspicion for malignancy. However, the final diagnosis was a benign fibroadenoma

We demonstrated predictions of the lesion detector and video classifier in Additional files 2 and 3, and the representative frames and heatmaps of the two videos in Fig. 6. The visualized feature maps use different color to highlight different degrees of informative regions relevant to the AI predictions. To be specific, the red regions contribute to high malignancy probability, and the blue regions to a benign probability.

## Discussion

The diagnostic accuracy for breast cancer varies significantly due to the high operator dependence among diagnostician across different regions, particularly in areas with unbalanced economic development [34]. In this work, we presented a DL model based on freehand US videos encompassing the whole lesion. The diagnostic accuracy of this model surpassed that of the static 2D-ResNet50 model and was comparable to the expertise of experienced radiologists in leading hospitals in China. Our model exhibited consistent and robust performance in external validation datasets across diverse hospital settings, patient age, lesion size, and BI-RADS category subgroups. Moreover, it demonstrated the ability to enhance the performance of experienced radiologists, improving overall accuracy and specificity while achieving greater interobserver diagnostic consistency.

AI has undergone explosive growth within the fields of medicine, manifesting in various applications encompassing public health, clinical-trial performance, image analysis, medical information retrieval, and operational organization [35]. Notably, AI models have exhibited remarkably success in the interpretation of medical images, engaging in tasks such as quantification, workflow triage, and image enhancement [17]. Despite the approval of over 200 commercial radiology AI products, the widespread integration of AI into clinical practice presents both promising prospects and notable challenges [17, 36].

Compared to other radiology modalities, the operator-dependent and non-standardized nature of static ultrasonic image acquisition presents a significant challenge in the generalization of existing AI models. Zhao et al. [24] highlighted that the performance of DL-based CAD systems may be influenced by the degree of image standardization. In response to the subjectivity introduced by radiologist-selected static images, researchers have explored methods for automatically acquiring frames from US videos. Huang et al. [37] proposed a reinforcement learning-based framework capable of automatically extracting keyframes from breast videos. Similarly, Chen et al. [38] introduced the Feature Entropy Breast Network (FEBrNet) to auto-catch responsible frames during breast US scanning. However, whether using radiologist-selected images, automatically extracted keyframes or autonomously captured responsible frames, these approaches fail to capture the comprehensive morphological characteristics of tumors.

On the other hand, video-based AI models, designed to capitalize on both morphological and temporal context information during dynamic scanning, have been applied to various clinical tasks. Liu et al. [39] proposed a video-based DL model with adjacent frame perception (AFP), enhancing the real-time detection of thyroid nodules by aggregating semantically similar contextual features in the video. Zhang et al. [40] developed a DL-video model using a 3D variant of ResNet-18 for differential diagnosis of thyroid carcinoma, demonstrating superior diagnostic performance compared to the 2D-ResNet-18 network in validation cohorts (AUC: 0.923 vs. 0.864, respectively, *p* = 0.028). Xu et al. [41] proposed an attention-boosted BConvLSTM-based diagnostic models, capturing spatiotemporal information from US videos for classifying benign versus malignant liver masses. The LM-VNet, using only US videos, achieved AUC of 0.966 in the developing dataset and 0.901 in the external test dataset, outperforming models using only US images. Li et al. [28] demonstrated the effectiveness of a DL model in predicting axillary lymph node metastases using dynamic US videos in breast cancer patients. The temporal interlace network (TIN) excelled with 0.914 AUC surpassing the static ResNet-50 model (AUC: 0.856, *p* < 0.05). Consistent with these advancements, our dynamic WAUVE model using I3D exhibited significantly higher diagnostic performance than the classic 2D-ResNet50 network. Notably, breast US examinations involve the dynamic scanning process in real-world clinical practice, emphasizing the superior compatibility of video-based AI model with the workflow of clinical radiologist.

A recently published literature reported a DL-based classification model utilizing breast US dynamic videos, achieving AUC of 0.969 in the internal test set. However, limitations in this study include the exclusive use of a single US machine, operated by only one experienced radiologist with a slow and uniform sweep speed. Furthermore, the absence of an independent test set raises concerns regarding the model’s generalizability [42]. On the contrary, our study implemented a more flexible approach to video acquisition, aligning with individual habits in routine clinical workflows. Meanwhile, the US machines used in our training set encompassed a broad spectrum of models commonly found in Chinese hospitals. In addition, our patient populations were sourced from both general and tumor specialized hospitals, representing diverse demographic characteristics. The development dataset, designed to closely mirror real-world clinical practice, enhances the generalization of algorithms in new settings and reduces the gap between research and practical application.

As the stand-alone diagnostic performance of the WAUVE model proved comparable to that of experienced radiologist in the prospectively collected external validation datasets, it may be feasible to explore implementation strategies for training general or junior radiologists with AI support. Although the PPVs exhibited differences across different subgroups, this might be attributed to the distinct prevalence of breast cancer, the imbalance distribution of patient characteristics, and the interpretation of BI-RADS category by different radiologists with various experience levels [43].

The issue of inter- and intra- reader inconsistency, along with high false positive rate of breast US diagnosis, has always been widely discussed. The discrepancy can create confusion among breast surgeons, impeding decision-making and hindering effective communication between doctors and patients. Consequently, patients often undergo reproducible examinations across different medical facilities. And overdiagnosis can lead to unnecessary biopsies or surgeries, which pose a considerable physical and psychological burden and additional economical costs to patients. In the AI-assisted reader study, the discernible value of WAUVE was to help radiologists enhance consistency across overall cases and heighten specificity in challenging scenarios, which indicated that it holds the promise of avoiding unnecessary diagnostic interventions and reducing associated health costs. Differences in the degree of BI-RADS adjustment after AI assistance—such as the relatively high number of upgrades by Radiologist 2—may reflect the acceptance and confidence of different radiologists in AI diagnosis, individual diagnostic preferences or thresholds for suspicion, rather than a lack of clinical experience. Furthermore, the superiority of our AI system over radiologists is underscored in time-consuming cases (*p* = 0.03). This suggests the feasibility of relying on AI for the diagnosis of intricate cases, potentially saving substantial time and enhancing the efficiency of US examinations.

This study has several limitations. First, it is customary for radiologists to integrate imaging findings with clinical information, such as symptoms and risk factors, when characterizing lesions. Notably, our model does not encompass these variations, making a subtle distinction between our model and actual clinical workflows. Future enhancements could involve incorporating the clinical information into the model to optimize diagnostic efficiency. Second, our reader study did not utilize bounding boxes and heatmaps. This omission was deliberate, recognizing that interpreting dynamic heatmaps could be time-exhausting for experienced radiologists, potentially affecting diagnostic efficiency. However, the integration of bounding boxes and heatmaps, if presented in real-time, could aid radiologists in locating and identifying the representative characteristics of the lesion. This has the potential to contribute to further improvements in AI-assisted diagnostic performance. Third, our study lacks videos depicting normal breasts. Consequently, it remains to be ascertained whether the model might misdiagnose normal breast tissues, accounting for different ages and physiological phases, as masses. The false positive value of the model’s automatic identification of lesions warrants further investigation. Finally, for the external validation test, videos from two hospitals were exclusively obtained using Philips machines. Despite this limitation, the validity of our model’s results is upheld since videos from Philips machines constituted only 4.6% of our training dataset. Ongoing examinations involve videos obtained using machines from multiple vendors to ensure a more comprehensive assessment.

In conclusion, our dynamic model demonstrated exceptional proficiency in assessing breast cancer under non-standardized B-mode ultrasound scanning conditions, facilitated the diagnostic performance of experienced radiologists, and improved diagnostic consistency and specificity for difficult cases. This clinically applicable AI system, designed to align with clinical US examinations, holds promise for integration into future breast US practices, potentially enhancing overall workflows.

## Supplementary Information

Additional file 1.

Additional file 2.

Additional file 3.

## Data Availability

All data generated or analyzed during this study are included in this article and its supplementary information files. The raw datasets from four hospitals are not publicly available use due to patient privacy, but some data can be available for academic purpose from the corresponding author on reasonable request, subject to permission from the institutional review board of the hospitals.

## Abbreviations

AFP: Adjacent frame perception
AI: Artificial intelligence
AUC: Area under the receiver operating characteristic curve
BI-RADS: Breast Imaging Reporting and Data System
CAD: Computer-aided diagnosis
CAM: Class activation map
CI: Confidence interval
DL: Deep learning
FDA: Food and Drug Administration
FPN: Feature pyramid network
GAP: Global average pooling
I3D: Inflated 3D ConvNet
ICC: Interclass correlation coefficient
NCC: National Cancer Center
NPV: Negative predictive value
PPV: Positive predictive value
PUMCH: Peking Union Medical College Hospital
ROC: Receiver operating characteristic curve
ROI: Region-of-interest
TIN: Temporal interlace network
US: Ultrasound
WAUVE: Whole-lesion-aware network based on freehand ultrasound video
