# Supplementary material for: Whole-lesion-aware network based on freehand ultrasound video for breast cancer assessment: a prospective multicenter study
Source: Cancer Imaging. 2025 Jun 16;25:75. doi: 10.1186/s40644-025-00892-y (PMC12172377; doi:10.1186/s40644-025-00892-y)
Supplement: Supplementary file 1 — Additional file 1. [file 40644_2025_892_MOESM1_ESM.docx]

**Whole-lesion-aware network based on freehand ultrasound video for breast cancer assessment: a prospective multicenter study**

**Supplementary materials**

1. A detailed description of our WAUVE model development
2. The construction of static 2D-ResNet-50 model
3. The construction of dynamic TimeSformer model
4. Supplementary Tables

**1.The WAUVE model development**

*1.1 Lesion detector*

As we all know, lesions typically appear in a limited spatial portion of a video. The large redundant background regions rendered it more difficult for the model to analyze specific lesions. Therefore, we first employed a lesion detector to eliminate background regions and to ensure the classification model concentrated more on the lesion areas. We adopted the Faster R-CNN^[1]^ with ResNet-34 backbone^[2]^ as the lesion detection network, and the feature pyramid network (FPN)^[3]^ was used to enhance the lesion detection capabilities on different scales. More specifically, the detection backbone mainly consists of four parts. The first part contains the convolutional layers of ResNet-34 that are used to extract features from each image, where residual blocks with skip connection and different scales in the pyramid network are used to ensure better feature extraction and fusion representation. The second part named the region proposal network (RPN), is a fully convolutional network that is used to generate a set of rectangular object proposals for lesions in each image. For each sliding-window location, nine reference proposals (anchors) are generated and used for the classification (distinguishing between positive and negative samples) and regression (correcting the coordinates of proposal boxes) tasks. In this process, the multi-task loss is designed to constrain the whole learning process of RPN, for an anchor box $i$, the loss function can be defined as:

$L\left(p_{i},t_{i}\right)=L_{cls}\left(p_{i},p_{i}^{*}\right)+\lambda p_{i}^{*}L_{reg}\left(t_{i},t_{i}^{*}\right)$, Eq. (A.1)

Where $p_{i}$is the predicted probability of anchor $i$, $p_{i}^{*}$is set to 1 once the anchor is labeled as positive or 0 if negative. Also, $t_{i}=\left\{t_{x},t_{y},t_{w},t_{h}\right\}$represents the coordinates of the predicted anchor box, and$t_{i}^{*}=\left\{t_{x}^{*},t_{y}^{*},t_{w}^{*},t_{h}^{*}\right\}$ represents the coordinates of the ground truth box labeled by radiologists. In addition, $L_{cls}$ and $L_{reg}$ represent the classification and regression loss functions, respectively. As shown in Eq. (A.2), (A.3) and (A.4), softmax loss between two categories (lesions and not lesions) is computed as the classification loss, while the smooth-L1 loss *R* is computed as the regression loss between the predicted coordinates with the ground truth one.

$L_{cls}\left(p_{i},p_{i}^{*}\right)=-\log\left[p_{i}p_{i}^{*}+\left(1-p_{i}\right)\left(1-p_{i}^{*}\right)\right]$, Eq. (A.2)

$L_{reg}\left(t_{i},t_{i}^{*}\right)=R\left(t_{i}-t_{i}^{*}\right)$, Eq. (A.3)

$R\left(x\right)=\left\{\begin{matrix} \begin{matrix} 0.5*x^{2} & \text{ }\left|x\right|<1 \end{matrix} \\ \begin{matrix} \left|x\right|-0.5 & otherwise \end{matrix} \end{matrix}\right.$. Eq. (A.4)

Additionally, the regression loss is only computed for all positive anchors ($p_{i}^{*}=1$). Loss balancing parameter $\lambda$ is set to 10, which means the model bias is more on the regression task.

The third part of the detection structure is ROI Pooling, which is used to combine the information from different scales and extract features from proposals. Based on RPN and ROI Pooling, the last part aims to use feature maps of proposals for classification and regression tasks, by which the final boxes of lesions with the correct category can be obtained for each image. It should be mentioned that the same loss function, used in the RPN, was adopted for the whole classification and regression tasks. Nevertheless, different from that in RPN, the input for the classification and regression head is the proposals generated from RPN. During the training process, the detection network is constrained by these two kinds of classification and regression loss functions.

To supervise the Faster R-CNN network, the bounding boxes of lesions were annotated by experienced radiologists in the development dataset. Before training process, we first extracted useful information from the original videos, in which only image areas in each video are preserved and all other areas are cropped. In this way, the average frame size of all videos is 562×857. During training, we adopted the Adam optimizer with a batch size of 128 frames and set the initial learning rate to 0.0004. The weights of ResNet-34 were initialized from the ImageNet pre-training. We trained the model for 50,000 iterations and decreased the learning rate by 0.1× at 30,000 and 40,000 iterations. During inference, we applied a simple tracking procedure to associate lesion boxes across adjacent frames into the same track. Specifically, for the boxes in the $t$ th frame in the video, we computed their Intersection over Union (IoU) with boxes in the $(t+1)$th frame. Boxes with IoU > 0.3 were assigned to the same track.

*1.2 Video classifier*

The video classifier predicted the cancer risk probability by aggregating the predictions of a series of video clips with eight frames. To create the video clips, for each lesion track, images within the ROI regions were firstly cropped. More specifically, to include contextual information, we expanded the ROI region by 30 pixels before cropping. Then, we grouped each adjacent eight frames sequentially in chronological order in a track and predicted the malignancy probability for each clip. For simplicity, tracks shorter than eight frames, as well as any extra ROIs at the end of a track that that did not contain enough frames to form an eight-frame clip, were excluded from the calculations of malignancy probability. The malignancy probability for each clip is denoted as $P_{i} (i\in[1, N])$, where $N$ is the number of video clips. Finally, the aggregated cancer risk of the video ${(P}_{A})$ was summarized by taking the average of all the clip results in the video.

The video classifier was instantiated using the Inflated 3D ConvNet (I3D)^[4]^ with ResNet-50 backbone^[2]^ in WAUVE. As a state-of-the-art video classification model, I3D has been widely adopted and performed well in natural video classification tasks on different datasets.^[5,6]^ Compared with the 2D classification structure, I3D has two main changes. In view of network structure, I3D simply converts the 2D classification models into 3D ConvNets to leverage the temporal information. In particular, all filters and pooling kernels in 2D architecture are inflated to add a temporal dimension. Thus, the original N×N filters in 2D structure are modified to N×N×N. Additionally, to leverage the pre-trained parameters, I3D also bootstraps the 3D filters from 2D ones. Concretely, the weights of 2D filters are firstly repeated by N times along the time dimensions, and then rescaled by dividing N. In this way, the 2D weights can be loaded by the 3D classification network structure and better temporal features can be extracted.

Based on the two aforementioned changes, we modified all filters in ResNet-50 structure to construct the 3D ConvNets, leveraged the strong model weights trained from the Kinetics 400 dataset^[7]^ and fine-tuned the model using the training set of the development US dataset. For each video, its *N* eight-frame clips are injected into *N* video classifiers, respectively, where the binary cross-entropy loss (shown in Eq. (A.5)) is computed to ensure the learning process for each classifier.

. Eq. (A.5)

Here, $p_{i}$ and $\hat{p}_{i}$ are the predicted and ground truth labels for each clip. After obtaining the predicted malignancy probability for each clip, the final malignancy score $S$ for each video can be calculated as follows:

$S=\frac{1}{N}\sum_{i=1}^{N} p_{i}$. Eq. (A.6)

During the training process of the video classifier, we used the SGD optimizer with a batch size of 64 video clips. The learning rate was initially set to 5×10^-2^ and decayed by 0.1 every 5 epochs. The total training duration was 12 epochs. In particular, 5-fold cross-validation is employed to search proper hyperparameters of the model, by which the influence of outliers can be minimized to guarantee the good generalization of our model.

*1.3 Heatmap generation and model interpretability*

For the training process, the GAP layer averaged the feature maps generated by the last convolutional layer, then the sum of these results was used to compute the final predicted score for each video clip. Similarly, the weighted sum of these feature maps was computed as the class activation map (CAM)^[8]^ or heatmap that emphasizes important areas in each image frame. As illustrated in Eq. (A.7), the CAM for the category $c$ was computed by using $f_{k}\left(x,y\right)$, the activation of unit $k$ at spatial location $\left(x,y\right)$, times with its corresponding weight $w_{k}^{c}$. In particular,$w_{k}^{c}$ represents the weights for the category $c$.

$M_{c}=\sum_{k} w_{k}^{c}f_{k}\left(x,y\right)$. Eq. (A.7)

In particular, for each video clip, the output feature maps of the last convolutional layer were C×4×H×W, where C, H and W represent the channel, width and height of the feature map, respectively. To obtain heatmaps for the eight static frame images in each clip, the nearest neighbor interpolation method was used to find the corresponding frame and generate the CAM for the target frame.

*1.4 Code availability*

The two modules in WAUVE were implemented by the publicly available deep learning packages and PyTorch framework. We used the Faster R-CNN in Detectron2 (https://github.com/facebookresearch/detectron2) in the lesion detector. The I3D model is from the SlowFast repository (https://github.com/facebookresearch/SlowFast).

**2.The static 2D-ResNet-50 model construction**

For the construction of static model, the lesion detector is the same with the dynamic model, while the classifier part is very different from the dynamic one. Concretely, the training set in the static model consists of two-view key frames (i.e., transverse and longitudinal views) ROI, where the ROI crop method that was used in the dynamic model is also used to generate ROI images for the static model. During the training process, the classifier in the static model was with the ImageNet pretrained ResNet-50 structure, and the softmax cross entropy loss function was used to constrain the whole learning process. The static model was trained for 12 epochs with the batch size of 16. In addition, the SGD optimizer was used in the static model and the learning rate was set to 0.005.

1. **The dynamic TimeSformer model construction**

To further verify the effect of our classifier backbone (i.e., I3D), we also performed comparison experiments with another method, TimeSformer.^[9]^ As a famous video understanding framework, TimeSformer inherits the architecture of ViT, and separates temporal attention and spatial attention modules in each block. During the training process, TimeSformer is utilized as the classifier backbone instead of the original I3D model, and the pre-trained model on K400 dataset^[7]^ is utilized to initialize the network parameters. The size of input images is 224×224, and the learning rate is initialized to 1×10^-3^. Additionally, all other training protocols are set as the same with that in I3D model.

As I3D surpasses TimeSformer in model efficiency, evidenced by its smaller size (139M vs. 485M parameters) and lower computational demand (44 vs. 380 GFLOPs), this makes I3D faster for training and inference, particularly beneficial in resource-limited clinical settings. Therefore, under the similar diagnostic performance, I3D was adopted as the classifier backbone of WAUVE.

**References**

1. Ren S, He K, Girshick R, Sun J (2017) Faster R-CNN: Towards Real-Time Object Detection with Region Proposal Networks. IEEE Trans Pattern Anal Mach Intell 39:1137-49.

2. He K, Zhang X, Ren S, Sun J (2016) Deep Residual Learning for Image Recognition. IEEE Conference on Computer Vision and Pattern Recognition (CVPR). DOI: 10.1109/CVPR.2016.90.

3. Lin TY, Dollar P, Girshick R, He K, Hariharan B, Belongis S (2017) Feature pyramid networks for object detection. IEEE Conference on Computer Vision and Pattern Recognition (CVPR). DOI: 10.1109/CVPR.2017.106.

4. Carreira J, Zisserman A (2017) Quo vadis, action recognition? a new model and the kinetics dataset. IEEE Conference on Computer Vision and Pattern Recognition (CVPR). DOI: 10.1109/CVPR.2017.502.

5. Kuehne H, Jhuang H, Garrote E, Poggio T, Serre T (2011) HMDB: a large video database for human motion recognition. International Conference on Computer Vision (ICCV). DOI: 10.1109/ICCV.2011.6126543.

6. Soomro K, Zamir AR, Shah M (2012) UCF101: A Dataset of 101 Human Actions Classes From Videos in The Wild. arxiv:12120402[csCV].

7. Kay W, Carreira J, Simonyan K, et al (2017) The Kinetics Human Action Video Dataset. arxiv:170506950[csCV].

8. Zhou B, Khosla A, Lapedriza A, Oliva A, Torralba A (2016) Learning Deep Features for Discriminative Localization. IEEE Conference on Computer Vision and Pattern Recognition (CVPR). DOI: 10.1109/CVPR.2016.319.

9. Bertasius G, Wang H, Torresani L (2021) Is Space-Time Attention All You Need for Video Understanding? arxiv:210205095[csCV].

**Supplementary Table 1. Sensitivity and specificity comparison of radiologists with and without AI assistance for different evaluation time intervals**

|  | Evaluation time (s) ^*^ | | | -10.45 - 12.67 | 13.03 - 26.03 | 26.08 - 95.49 |
| --- | --- | --- | --- | --- | --- | --- |
| Sensitivity(%,95%CI) | | w/o AI^†^ | 1.00 (1.00-1.00) | | 0.97 (0.93-0.99) | 0.98 (0.94-0.99) |
|  |  | w AI^‡^ | 1.00 (1.00-1.00) | | 0.97 (0.93-0.99) | 0.99 (0.96-1.00) |
| *p* value | | | 1.00 | | 1.00 | 1.00 |
| Specificity(%,95%CI) | | w/o AI^†^ | 0.64 (0.52-0.75) | | 0.52 (0.42-0.61) | 0.40 (0.31-0.49) |
|  |  | w AI^‡^ | 0.69 (0.58-0.80) | | 0.60 (0.50-0.69) | 0.46 (0.37-0.55) |
| *p* value | | | 0.30 | | 0.18 | 0.027 |

^*^ Diagnostic time of radiologists without AI assistance minus the duration of each breast lesion video. ^†^ Without AI assistance. ^‡^ With AI assistance
